# Knowledge and Myths about Eating Disorders in a German Adolescent Sample: A Preliminary Investigation

**DOI:** 10.3390/ijerph19116861

**Published:** 2022-06-03

**Authors:** Johannes Feldhege, Sally Bilic, Kathina Ali, Daniel B. Fassnacht, Markus Moessner, Louise M. Farrer, Kathleen M. Griffiths, Stephanie Bauer

**Affiliations:** 1 Center for Psychotherapy Research, Heidelberg University Hospital, 69115 Heidelberg, Germany; sally.bilic@mail.de (S.B.); markus.moessner@med.uni-heidelberg.de (M.M.); stephanie.bauer@med.uni-heidelberg.de (S.B.); 2 College of Education, Psychology and Social Work, Flinders University, Adelaide 5042, Australia; kathina.ali@flinders.edu.au (K.A.); dan.fassnacht@flinders.edu.au (D.B.F.); 3 Órama Institute for Mental Health and Wellbeing, Flinders University, Adelaide 5042, Australia; 4 Research School of Psychology, Australian National University, Canberra 2601, Australia; kathy.griffiths@anu.edu.au; 5 Centre for Mental Health Research, Australian National University, Canberra 2601, Australia; louise.farrer@anu.edu.au; 6 College of Health & Medicine, Australian National University, Canberra 2601, Australia

**Keywords:** eating disorder, mental health literacy, eating disorder literacy, stigma, help-seeking

## Abstract

Eating disorder mental health literacy (ED-MHL) refers to knowledge about the symptoms, causes, and treatment of eating disorders (EDs) and is an important factor in people’s attitudes towards individuals with EDs and help-seeking for EDs. Associations between ED-MHL, stigma, ED symptomatology, and gender were investigated in a sample of N = 194 German high school students. Knowledge and myths about EDs were assessed with 18 factual statements about EDs and agreement/disagreement with common myths about ED. Students also completed the Universal Stigma Scale (USS), the Weight Concerns Scale (WCS), and demographic items. Students judged M = 8.39 (SD = 3.40) statements correctly, while the average agreement with all ED myths was low (M = 0.19, SD = 0.14). Greater ED-MHL was associated with lower stigmatization of EDs. Male participants were less knowledgeable and more likely to agree with the ED myths. Participants displayed moderate ED-MHL; however, certain aspects such as ED risk factors or symptoms of specific disorders such as anorexia nervosa and bulimia nervosa were less well known. These results can inform the design of future MHL interventions for adolescents.

## 1. Introduction

Poor eating disorder mental health literacy (ED-MHL) has been recognized as an important barrier to treatment seeking for eating disorders (EDs) in adolescents and adults [1,2,3]. According to a definition by Jorm and colleagues [4], MHL is a concept that encompasses (1) the ability to recognize specific mental disorders or different types of psychological distress, (2) the knowledge about risk factors and causes of mental illness, (3) the knowledge about self-help interventions and available professional help, (4) the knowledge about how to look for information on mental health, as well as (5) attitudes which facilitate recognition and appropriate help-seeking. One way that poor ED-MHL prevents treatment seeking is through the failure to realize the severity of symptoms or conditions that require treatment. Previous studies have found that adolescents’ recognition of EDs in themselves and others tends to be poor [5,6]. For example, many adolescents underestimate the severity of consequences associated with EDs while at the same time overestimating the prevalence of EDs [7]. Additionally, poor ED-MHL may act as a barrier for treatment seeking through a lack of knowledge about available treatments and a perception of the inadequacy of professional help [1]. Adolescents are skeptical about the benefits of seeing a mental health professional and prefer self-help interventions or support from relatives or close friends [8]. Finally, poor MHL is also related to greater stigmatization of EDs, which in itself may act as a barrier for treatment seeking [1,9]. Therefore, many interventions to reduce stigma contain educational components designed to replace stereotypes with factually correct information [10].

While a number of different measures have been used to assess knowledge about general mental health, as well as specific mental disorders [11], there are generally two main approaches for assessing MHL: diagnostic vignettes and fact-based tests [12]. The most common method of assessing ED-MHL involves providing vignettes describing individuals with ED symptoms, and asking individuals to identify the condition experienced by the person in the vignette (e.g., [13]). A number of studies target additional aspects of ED-MHL including, for example, knowledge about helpful interventions [8], knowledge about causes and risk factors [14], and knowledge about severity and prevalence of EDs [15]. Existing vignette-based measures, however, have some methodological limitations: in their basic form, they do not allow for the calculation of a MHL score, which could be used to compare the level of MHL within and between individuals [12]. Additionally, participants’ answers to further questions depend on their prior recognition of the mental disorder in the vignette. If participants misinterpret the ED in the vignette as something else, e.g., stress or unhealthy nutrition, they could attribute other causes or recommend other interventions for these conditions. Furthermore, the response format used in ED-MHL vignette tests can make answering and analyzing these measures cumbersome and time consuming.

In order to address these limitations, we used two newly developed, not yet validated questionnaires [16] and applied them in the present preliminary study in a sample of adolescents. The first was a fact-based ED literacy measure that can be answered and analyzed quickly and does not depend on participants correctly recognizing the ED in a vignette. The second questionnaire assessed the prevalence of beliefs in common myths about EDs (for further information on the development and validation of both questionnaires see [16]).

There are a number of demographic and ED-related factors that might be relevant to how much a person knows about EDs. In addition to stigma, an individual’s level of ED symptoms might influence MHL. This is especially apparent in the poor self-recognition of ED behaviors in adolescent girls with ED symptoms [5,8]. ED-MHL also differs by gender. Female adolescents have greater knowledge about EDs than male adolescents [17], young men are less likely to recognize EDs, and consider them to be less severe than young women [18].

For many individuals with EDs, the onset of their symptoms typically occurs in adolescence [19]. At the same time, only a minority of individuals ever receive treatment for EDs [20], and younger individuals are less likely to seek treatment [21,22]. Therefore, research into adolescents’ barriers for treatment-seeking, such as ED-MHL, is essential to inform prevention and early intervention efforts. However, there are few studies investigating ED-MHL in both male and female adolescents. Instead, most studies focused either on adults or on female adolescents only (e.g., [8,13]). Therefore, in the present study, we investigated ED-MHL in a sample of German male and female adolescents to address the lack of research in this area.

There were two aims for this study: Firstly, to establish the level of ED-MHL and agreement with ED myths. Secondly, to explore the relationships between ED-MHL, ED myths and demographic, and ED-related variables. Specifically, we considered participants’ level of ED symptoms and stigmatizing attitudes towards individuals with ED. Additionally, we investigated whether ED-MHL and agreement with ED myths varied with participants’ gender.

## 2. Materials and Methods

### 2.1. Participants

The study was conducted in a “Gymnasium”, a type of high school that focuses on preparing students for higher education at university, in North Rhine-Westphalia during a school visit in the context of the EU project ProYouth, an online ED prevention, and early intervention program for young people available in several languages [23,24]. Students in grades 7–10 were eligible to participate in the study. Informed consent to complete pen-paper questionnaires was obtained from both the students and their parents. Ethical approval was obtained from the human ethics committee of the Medical Faculty of the University of Heidelberg (Protocol No. S-465/2017). Participants completed the questionnaires during class time. Demographic information of the sample can be found in Table 1.

### 2.2. Measures

#### 2.2.1. Eating Disorder Mental Health Literacy and Eating Disorder Myths

To measure the level of ED-MHL, 18 factual statements about symptoms, risk factors and causes, help-seeking, treatment, and recovery of EDs broadly, as well as anorexia nervosa and bulimia nervosa specifically, were used [16]. The statements have been developed based on the Depression Literacy Questionnaire [25,26], and are answered with ‘true’, ‘false’, or ‘don’t know’. Eight of the statements were formulated with ‘true’ as the correct answer, while ten had ‘false’ as the correct answer. Respondents scored one point for each correct response, with higher scores indicating better eating disorder mental health literacy.

The questionnaire assessing beliefs in ED myths has its origins in the “Nine Truths about Eating Disorders”, statements representing state-of the-art knowledge about EDs [27]. These truths were translated into 30 languages and disseminated widely in order to combat common misconceptions about EDs and counter stigma. The 11 items are based on the original wording of the myths about EDs (e.g., “You can tell by looking at someone that they have an eating disorder” or “Eating disorders are a lifestyle choice, not serious illnesses”); two additional items were added from the National Eating Disorder Collaboration’s myths (https://nedc.com.au/eating-disorders/eating-disorders-explained/myth/, accessed on 1 June 2022), one about EDs as attention seeking or a phase and a second one about diets as a normal part of life [16]. All items were rated on a 4-point Likert scale (1 = strongly disagree, 2 = disagree, 3 = agree, 4 = strongly agree). Participants’ responses were dichotomized into disagreement (answer options 1, 2) and agreement (answer options 3, 4) and summed to provide an ED Myths score for each participant. 

We report the average number of correct and “don’t know” answers for ED-MHL as well as the ED myths score. Furthermore, we also report values for each item separately as they cover a number of different areas, and we consider the level of knowledge or false belief in a myth in each separate area in our participants as informative. ED-MHL statements and myths were originally developed in English [16], then translated into German, and finally back-translated into English to ensure equivalency in both languages. The items’ wording in English, along with their mean values, can be found in Table 2 and Table 3, respectively. German versions of both sets of items can be found in Tables S1 and S2 in the Supplement. Internal consistencies of the ED MHL scale and the ED Myths scores were α = 0.79 and α = 0.45, respectively. 

#### 2.2.2. Weight and Shape Concerns

Participants’ concerns about their body weight and shape were assessed using the Weight Concerns Scale (WCS) [28]. A cutoff of 57 was used to indicate a risk of developing an ED [29]. Additionally, two self-report items assessing body weight and height were included to allow for the calculation of the Body Mass Index (BMI). Participants were also asked whether they had ever been diagnosed with or treated for an ED. The WCS had high internal consistency with a Cronbach’s Alpha of α = 0.82.

#### 2.2.3. Eating Disorder Stigma

The Universal Stigma Scale (USS; [30]) was used to measure ED stigma with eleven items on a 5-point Likert scale (1 = strongly disagree, 5 = strongly agree). The scale has two subscales: blame/personal responsibility and impairment/distrust, each consisting of five and six items, respectively. The internal consistency in the current sample, as assessed with Cronbach’s Alpha, was α = 0.78 for the blame/personal responsibility subscale and α = 0.83 for the impairment/distrust subscale.

### 2.3. Statistical Analysis

The relationships between all variables were explored with Pearson correlation coefficients. Differences between male and female participants, in agreement with ED myths and correct answers to the ED-MHL statements, were tested with independent t-tests. All statistical analyses were carried out using the IBM SPSS Statistics program.

## 3. Results

In total, 223 students participated in the study. Out of these, 29 (12.4%) students were excluded from the analysis as they had missing values for age and/or gender or more than 25% missing values in the ED-MHL statements or the ED myths items. All analyses were conducted with the remaining 194 participants. Despite removing participants with a high number of missing values in the measures of interest, there were still small numbers of missing values in most of the items and questionnaires. We report the number of participants that had non-missing values for each item and scale in Table 1, Table 2 and Table 3. For the ED Myths score, and the correct answers to the ED-MHL statements, we counted only the non-missing items that matched their respective criteria. For the “don’t know” answers we counted the number of times a participant chose that option as well as the number of times they left an item blank.

The sample’s demographic information and descriptive statistics can be found in Table 1. Seventeen participants (8.8%) had a BMI below the 10th percentile for their age and gender. Twelve participants (6.2%) had a WCS score above 57. One participant indicated that they had been diagnosed with an ED (Binge Eating Disorder) and that they were currently in treatment. For the ED-MHL scale, five participants (2.6%) did not answer any of the statements correctly, while one participant answered all statements correctly. Table 2 displays the percentage of participants agreeing with each ED myth, and Table 3 displays the percentage of correct and “don’t know” answers for each statement separately.

Correlations between age, USS, WCS, the number of ED myths agreed to, and number of correct EDL statements can be found in Table 4. Participants’ age had significant but small correlations with the USS distrust scale and the number of agreed myths. The latter also showed a small, negative correlation with the percentage of correctly answered statements. ED myths agreement was significantly and positively correlated with both USS stigma scales, while the number of correct answers to the ED-MHL statements was negatively related to the USS blame/personal responsibility scale. There was a significant difference between gender in agreement with ED myths: male participants M = 0.21 (SD = 0.16), female participants M = 0.17 (SD = 0.12); *t*(159.63) = 2.16, *p* = 0.03). Additionally, the number of correct answers differed significantly between the two genders (male participants M = 7.56 (SD = 3.28), female participants M = 9.09 (SD = 3.35); *t*(191) = −3.20, *p* < 0.01).

## 4. Discussion

In the present preliminary study, we investigated ED mental health literacy and agreement with ED myths in a sample of adolescents between the ages of 12 and 17 recruited from a German high school. Additionally, we examined how factors such as ED stigma, ED symptomatology, and gender were related to ED-MHL and endorsement of ED myths. As ED-MHL and ED Myths were assessed with new, not yet validated measures, our results should be interpreted with caution. 

Generally, the fact-based statements suggest moderate ED-MHL, with participants answering on average eight out of eighteen statements correctly. Additionally, participants answered “don’t know” on a third of the statements. The number of correct answers was not spread equally among all statements. Knowledge about treatment effectiveness and recovery from EDs could be considered relatively high, with more than 70% of students answering items such as “Effective treatment for eating disorders is available” and “If people seek help for an eating disorder early, they will recover faster” correctly. In contrast, ED-MHL concerning risk factors for EDs appears relatively low (e.g., the item “Genes do not play a role in the development of eating disorders”). Additionally, specific knowledge about bulimia nervosa and anorexia nervosa seems limited with respective items (e.g., “People have to vomit to have bulimia nervosa” and “Males can develop anorexia nervosa, but not bulimia nervosa”), indicating the lowest percentages of correct answers with 15–35%. These values are lower than the number of correct answers for definitions of anorexia nervosa and bulimia nervosa in another study [17].

Participants’ average endorsement of two out of eleven myths suggests a low agreement with ED myths in the present sample. This is much lower than would be expected if participants answered the items randomly. The myth with the highest level of agreement was about how a person with an ED can be recognized by their appearance, which might be influenced by the stereotypical image of emaciated individuals with anorexia nervosa portrayed in the media. However, eating disorders are present at all levels of body weight and somatic effects of eating disorders are not always visible [27]. 

The correlational analysis showed that greater knowledge, i.e., a higher number of correctly answered ED-MHL statements and lower agreement with myths, could be related to lower stigmatization. The same relationship can be found in a number of studies (e.g., [9]), indicating that knowledge is an important factor in reducing stigma.

Stronger agreement with ED myths was associated with lower ED-MHL in our study. However, the association was weak, suggesting that agreement with ED myths might not just be a sign of an absence of ED-MHL, but could capture a separate construct from ED-MHL.

Somewhat surprisingly, we found small, positive correlations of age with the USS distrust scale and the number of agreed ED myths. Older students were more likely to distrust individuals with EDs and might endorse more myths about EDs. Studies examining the relationship between age, stigma, and ED literacy in adolescents are few but their results indicate no differences between younger and older adolescents (e.g., [5,6]). 

Male participants in our study were more likely to endorse ED myths and displayed lower ED-MHL. These results are in line with another study that demonstrated reduced ED knowledge in adolescent boys than girls [17]. Even though the prevalence of EDs in male adolescents is lower than in females [31], the observed lack of ED-MHL and mistaken beliefs in ED myths in male adolescents in the present study could point to a need for future ED-MHL interventions to specifically target this group so that they can search out help if they themselves suffer from an ED in the future or facilitate help-seeking in friends or family members. 

### Limitations

A first limitation is that detailed information on the factor validity of the ED-MHL and ED Myths scales could not be provided in the present study. The absence of any validated scales assessing knowledge and misconceptions about EDs led to the development of these new measures [16]. The present study did not undertake psychometric analyses of the factor validity of the new scales. Such an analysis will be presented in a forthcoming separate paper based on data from a suitably large sample [16]. However, the scales have high face validity as they assess knowledge and myths about EDs. The ED Myths scale can also be said to have high content validity as its items cover the most common misconceptions of EDs identified by leading experts in the field [3,27]. Furthermore, our results present some evidence of construct validity with significant associations between stigma, ED Myths, and ED-MHL, as well as ED-MHL and gender.

Secondly, the internal consistency of the ED Myths score was low at α = 0.45. However, it is important to note that the ED Myths items were not developed to form a unified scale but rather that they were based on a diverse set of common misconceptions about EDs. It is therefore possible that participants would strongly agree with some of the myths while rejecting others, leading to a low internal consistency.

Finally, the current sample did not experience elevated levels of ED symptomatology and only a few adolescents were identified as at-risk for the development of an ED. While the average BMI value was near the lower bound of the normal weight class, relatively few participants had a BMI that was below the 10th percentile for their age and gender. Furthermore, the average WCS score was lower and fewer participants scored above the cutoff of 57 points than in comparable school samples [32]. Therefore, it remains unclear whether the results can be generalized to more severely affected populations. Furthermore, students were recruited from a single school with a generally high level of education. Our results on ED-MHL may not be representative for adolescents in Germany attending other types of high schools, as adolescents from German schools of a lower educational level had problems in recognizing an ED in a vignette and identifying its causes [6]. This suggests that future studies should examine adolescent’s ED-MHL in different types of schools with varying levels of education. 

## 5. Conclusions

The present study is one of the few that has concurrently investigated ED-MHL in male and female adolescents. Overall, we found indicators for moderate knowledge about EDs and more specifically, extensive knowledge about treatment efficacy and recovery from EDs among the adolescents in our sample. The new questionnaires measure factual knowledge about EDs and agreement with ED myths in an efficient and objective manner. The present results suggest that interventions targeting male adolescents and focusing on those areas where participants were less knowledgeable, such as risk factors for EDs or symptoms specific to certain EDs, could lead to the greatest gains in knowledge. As such, male adolescents could become better equipped to identify ED symptoms in themselves and support others with EDs in their help-seeking. Furthermore, a better understanding of risk factors or specific ED symptoms could lead to better early recognition of the warning signs of EDs in all adolescents and timely help-seeking to prevent a chronic course, which is common in EDs. However, there is a need to validate the new measures, replicate our findings, and investigate other groups affected by EDs or at risk for development of EDs.

## Figures and Tables

**Table 1:** Demographics, Body Mass Index (BMI), Weight Concerns Scale (WCS), Universal Stigma Scales (USS), Eating Disorder (ED), Myths score, and Number of Correct ED Mental Health Literacy (ED-MHL) statements of N = 194 German Adolescents.

	*n* (%)	Mean (SD)	Range
Gender			
Male	91 (46.9%)		
Female	102 (52.6%)		
Other	1 (0.5%)		
Age	194 (100%)	14.16 (1.33)	12–17
BMI	177 (91.2%)	19.42 (2.34)	13.98–29.30
WCS	178 (91.8%)	22.61(18.80)	0–90
USS blame/personal responsibility	188 (96.9%)	2.16 (0.82)	1–5
USS impairment/distrust	188 (96.9%)	1.91 (0.81)	1–5
ED Myths Score	194 (100%)	0.19 (0.14)	0–1
N correct ED-MHL answers	194 (100%)	8.39 (3.40)	0–18
N ‘don’t know’ ED MHL answers	194 (100%)	6.88 (3.96)	0–18

Note. Means and SDs are reported for participants with non-missing values for each item or scale. BMI = Body Mass Index (kg/m^2^), WCS = Weight Concerns Scale; USS = Universal Stigma Scale. ED Myths Score: number of ED Myths items participants “agree” or “strongly agree” with.

**Table 2:** Text and percentage of participants who “agree” or “strongly agree” with the 11 ED myths.

	*n* (%)	“Agree” or “Strongly Agree” (%)
You can tell by looking at someone that they have an eating disorder.	189 (97.4%)	54.6
Families are to blame when people develop an eating disorder.	191 (98.5%)	13.4
An eating disorder diagnosis is not a health crisis that disrupts personal and family functioning.	185 (95.4%)	25.3
Eating disorders are a lifestyle choice, not serious illnesses.	194 (100%)	5.2
Eating disorders only affect white upper-middle class teenage girls and young women.	191 (98.5%)	4.6
Eating disorders do not carry an increased risk for suicide or medical complications.	186 (95.9%)	18.0
Only society and the media play a role in the development of eating disorders.	191 (98.5%)	19.1
Genes alone predict who will develop an eating disorder.	188 (96.9%)	9.3
Full recovery from an eating disorder is not possible.	185 (95.4%)	13.4
Eating disorders are a cry for attention or a person going ‘through a phase’.	192 (99.0%)	4.6
Dieting is a normal part of life.	188 (96.9%)	34.5

Note. Percentage of participants agreeing with an ED myth is based on the number of participants with non-missing values for each item.

**Table 3:** Text and percentages of correct and “don’t know” answers for the 18 ED-MHL statements.

	*n* (%)	Correct %	“Don’t Know” %
1. Effective treatment for eating disorders is available.	194 (100%)	81.4	16.5
2. Once someone has an eating disorder, recovery is very unlikely.	192 (99.0%)	74.5	17.7
3. Rapid weight loss in a short period of time can be a symptom of anorexia nervosa.	192 (99.0%)	35.9	55.2
4. Genes do not play a role in the development of eating disorders.	193 (99.5%)	26.4	46.1
5. Eating disorders have the highest mortality rate among all mental illnesses.	193 (99.5%)	35.2	51.8
6. Males can develop anorexia nervosa, but not bulimia nervosa.	193 (99.5%)	35.2	59.1
7. If people seek help for an eating disorder early, they will recover faster.	192 (99.0%)	74.5	20.3
8. People with bulimia nervosa can be slightly underweight, normal weight, or overweight.	193 (99.5%)	28.0	61.7
9. Most people have experienced binge eating at some point in their life.	191 (98.5%)	16,2	41.4
10. People have to vomit to have bulimia nervosa.	192 (99.0%)	19.8	47.4
11. It is common for a person with an eating disorder to also experience another mental illness, such as depression.	193 (99.5%)	56.0	33.7
12. People with eating disorders can stop the behavior if they want to.	190 (97.9%)	68.9	17.4
13. Eating disorders only affect adolescent girls and young adult women.	193 (99.5%)	78.2	19.2
14. Not everyone with an eating disorder needs to seek help.	191 (98.5%)	40.3	34.0
15. Eating disorders are simply caused by western cultural values of thinness.	194 (100%)	45.4	43.8
16. Rapid weight loss or being very underweight can affect your ability to think.	192 (99.0%)	37.5	52.1
17. A person with an eating disorder might find it difficult to ask for help from family and friends.	193 (99.5%)	76.2	17.1
18. People who have had an eating disorder will always worry about their weight, even if they have fully recovered.	194 (100%)	15.5	44.8

Note. Percentages of correct answers are based on the number of participants with non-missing values for each item. Percentage of “don’t know” answers include counts of users leaving the item blank.

**Table 4:** Correlations between USS scales Blame/responsibility and Impairment/distrust, WCS, ED Myths Score, and correct answers to ED-MHL statements.

	Age	USS Blame/Personal Responsibility	USS Impairment/Distrust	WCS	N Agreed ED Myths
Age					
USS Blame/personal responsibility	−0.08				
USS Impairment/distrust	0.18 *	0.50 **			
WCS	0.03	−0.21 **	−0.06		
N agreed ED myths	0.19 *	0.28 **	0.38 **	<0.00	
N correct ED-MHL answers	0.02	−0.31 **	−0.08	0.10	−0.22 **

Note. * *p* < 0.05. ** *p* < 0.01. WCS = Weight Concerns Scale; USS = Universal Stigma Scale.

## Data Availability

The data presented in this study are available on request from the corresponding author. The data are not publicly available due to legal and privacy issues.

## Supplementary Materials

The following supporting information can be downloaded at: https://www.mdpi.com/article/10.3390/ijerph19116861/s1, Table S1: German wording of ED Myths scale; Table S2: German wording of ED-MHL statements.

## References

[1] Ali K., Farrer L., Fassnacht D.B., Gulliver A., Bauer S., Griffiths K.M. (2017). Perceived barriers and facilitators towards help-seeking for eating disorders: A systematic review. Int. J. Eat. Disord..

[2] Innes N.T., Clough B.A., Casey L.M. (2017). Assessing treatment barriers in eating disorders: A systematic review. Eat. Disord..

[3] Ali K., Fassnacht D.B., Farrer L., Rieger E., Feldhege J., Moessner M., Griffiths K.M., Bauer S. (2020). What prevents young adults from seeking help? Barriers toward help-seeking for eating disorder symptomatology. Int. J. Eat. Disord..

[4] Jorm A.F., Korten A.E., Jacomb P.A., Christensen H., Rodgers B., Pollitt P. (1997). “Mental health literacy”: A survey of the public’s ability to recognise mental disorders and their beliefs about the effectiveness of treatment. Med. J. Aust..

[5] Gratwick-Sarll K., Bentley C., Harrison C., Mond J. (2016). Poor self-recognition of disordered eating among girls with bulimic-type eating disorders: Cause for concern?. Early Interv. Psychiatry.

[6] Feldhege J., Gulec H., Moessner M., Stieler C., van Stipelen J., Bauer S. (2022). Stigmatization and attitudes toward eating disorders: A comparison between native German adolescents, Turkish immigrant adolescents in Germany, and native Turkish adolescents. J. Ment. Health.

[7] Mond J.M., Marks P. (2007). Beliefs of adolescent girls concerning the severity and prevalence of bulimia nervosa*. Aust. J. Psychol.

[8] Mond J.M., Marks P., Hay P.J., Rodgers B., Kelly C., Owen C., Paxton S.J. (2007). Mental Health Literacy and Eating-Disordered Behavior: Beliefs of Adolescent Girls Concerning the Treatment of and Treatment-Seeking for Bulimia Nervosa. J. Youth Adolesc..

[9] Rodgers R.F., Paxton S.J., McLean S.A., Massey R., Mond J.M., Hay P.J., Rodgers B. (2015). Stigmatizing attitudes and beliefs toward bulimia nervosa: The importance of knowledge and eating disorder symptoms. J. Nerv. Ment. Dis..

[10] Doley J.R., Hart L.M., Stukas A.A., Petrovic K., Bouguettaya A., Paxton S.J. (2017). Interventions to reduce the stigma of eating disorders: A systematic review and meta-analysis. Int. J. Eat. Disord..

[11] Wei Y., McGrath P.J., Hayden J., Kutcher S. (2015). Mental health literacy measures evaluating knowledge, attitudes and help-seeking: A scoping review. BMC Psychiatry.

[12] O’Connor M., Casey L., Clough B. (2014). Measuring mental health literacy—A review of scale-based measures. J. Ment. Health.

[13] Mond J.M., Hay P.J., Paxton S.J., Rodgers B., Darby A., Nillson J., Quirk F., Owen C. (2010). Eating Disorders “Mental Health Literacy” in Low Risk, High Risk and Symptomatic Women: Implications for Health Promotion Programs. Eat. Disord..

[14] Mond J.M., Hay P.J., Rodgers B., Owen C., Beumont P.J.V. (2004). Beliefs of Women Concerning Causes and Risk Factors for Bulimia Nervosa. Aust. N. Z. J. Psychiatry.

[15] Mond J.M., Hay P.J., Rodgers B., Owen C., Beumont P.J.V. (2004). Beliefs of women concerning the severity and prevalence of bulimia nervosa. Soc. Psychiatry Psychiatr. Epidemiol..

[16] Ali K., Farrer L., Rieger E., Griffiths K.M., O’Shea A., Feldhege J., Moessner M., Bauer S., Fassnacht D. (2022). Eating disorder literacy among young adults in Australia.

[17] Napolitano F., Bencivenga F., Pompili E., Angelillo I.F. (2019). Assessment of Knowledge, Attitudes, and Behaviors toward Eating Disorders among Adolescents in Italy. Int. J. Environ. Res. Public Health.

[18] Mond J.M., Arrighi A. (2011). Gender differences in perceptions of the severity and prevalence of eating disorders. Early Interv. Psychiatry.

[19] Swanson S.A., Crow S.J., Le Grange D., Swendsen J., Merikangas K.R. (2011). Prevalence and correlates of eating disorders in adolescents. Results from the national comorbidity survey replication adolescent supplement. Arch. Gen. Psychiatry.

[20] Hart L.M., Granillo M.T., Jorm A.F., Paxton S.J. (2011). Unmet need for treatment in the eating disorders: A systematic review of eating disorder specific treatment seeking among community cases. Clin. Psychol. Rev..

[21] Forrest L.N., Smith A.R., Swanson S.A. (2017). Characteristics of seeking treatment among U.S. adolescents with eating disorders. Int. J. Eat. Disord..

[22] Bohrer B.K., Carroll I.A., Forbush K.T., Chen P.-Y. (2017). Treatment seeking for eating disorders: Results from a nationally representative study. Int. J. Eat. Disord..

[23] Bauer S., Papezova H., Chereches R., Caselli G., McLoughlin O., Szumska I., van Furth E., Ozer F., Moessner M. (2013). Advances in the prevention and early intervention of eating disorders: The potential of Internet-delivered approaches. Ment. Health Prev..

[24] Ali K., Fassnacht D.B., Farrer L.M., Rieger E., Moessner M., Bauer S., Griffiths K.M. (2022). Recruitment, adherence and attrition challenges in internet-based indicated prevention programs for eating disorders: Lessons learned from a randomised controlled trial of ProYouth OZ. J. Eat. Disord..

[25] Griffiths K.M., Christensen H., Jorm A.F., Evans K., Groves C. (2004). Effect of web-based depression literacy and cognitive–behavioural therapy interventions on stigmatising attitudes to depression: Randomised controlled trial. Br. J. Psychiatry.

[26] Gulliver A., Griffiths K.M., Christensen H., Mackinnon A., Calear A.L., Parsons A., Bennett K., Batterham P.J., Stanimirovic R. (2012). Internet-Based Interventions to Promote Mental Health Help-Seeking in Elite Athletes: An Exploratory Randomized Controlled Trial. J. Med. Internet Res..

[27] Schaumberg K., Welch E., Breithaupt L., Hübel C., Baker J.H., Munn-Chernoff M.A., Yilmaz Z., Ehrlich S., Mustelin L., Ghaderi A. (2017). The Science Behind the Academy for Eating Disorders’ Nine Truths About Eating Disorders. Eur. Eat. Disord. Rev..

[28] Killen J.D., Taylor C.B., Hayward C., Wilson D.M., Haydel K.F., Hammer L.D., Simmonds B., Robinson T.N., Litt I., Varady A. (1994). Pursuit of thinness and onset of eating disorder symptoms in a community sample of adolescent girls: A three-year prospective analysis. Int. J. Eat. Disord..

[29] Killen J.D., Taylor C.B., Hayward C., Haydel K.F., Wilson D.M., Hammer L., Kraemer H., Blair-Greiner A., Strachowski D. (1996). Weight concerns influence the development of eating disorders: A 4-year prospective study. J. Consult. Clin. Psychol..

[30] Ebneter D.S., Latner J.D. (2013). Stigmatizing attitudes differ across mental health disorders: A comparison of stigma across eating disorders, obesity, and major depressive disorder. J. Nerv. Ment. Dis..

[31] Hammerle F., Huss M., Ernst V., Bürger A. (2016). Thinking dimensional: Prevalence of DSM-5 early adolescent full syndrome, partial and subthreshold eating disorders in a cross-sectional survey in German schools. BMJ Open.

[32] Bauer S., Bilić S., Ozer F., Moessner M. (2020). Dissemination of an Internet-Based Program for the Prevention and Early Intervention in Eating Disorders. Z. Für Kinder- Und Jugendpsychiatrie Und Psychother..

