# Supplementary material for: Knowledge and Myths about Eating Disorders in a German Adolescent Sample: A Preliminary Investigation
Source: Int J Environ Res Public Health. 2022 Jun 3;19(11):6861. doi: 10.3390/ijerph19116861 (PMC9180431; doi:10.3390/ijerph19116861)
Supplement: Supplementary file 1 [file ijerph-19-06861-s001.zip › ijerph-1716706-supplementary.pdf]

Table S1. German wording of ED Myths scale.

1. Menschen mit Essstörungen sind selbst schuld an ihrem Problem.
2. Menschen mit Essstörungen könnten damit aufhören, wenn sie es wollten.
3. Essstörungen sind ein Zeichen von persönlicher Schwäche.
4. Menschen mit Essstörungen könnten sich zusammenreißen, wenn sie es nur wollten.
5. Essstörungen sind keine echten Krankheiten.
6. Menschen mit Essstörungen sind gefährlich.
7. Als Arbeitgeber würde ich niemanden einstellen, wenn ich wüsste, dass er/sie eine Essstörung hat.
8. Ich würde kein/e Politiker/in wählen, von dem/der ich wüsste, dass er/sie eine Essstörung hatte.
9. Es ist schwierig, mit Menschen mit Essstörungen zu reden.
10. Menschen mit Essstörungen sind unberechenbar.
11. Menschen mit Essstörungen sind weniger kompetent als andere.

Table S2. German wording of ED-MHL statements

1. Es gibt effektive Behandlungen für Essstörungen.
2. Es ist unwahrscheinlich, dass jemand mit einer Essstörung wieder gesund wird.
3. Ein starker Gewichtsverlust innerhalb kurzer Zeit kann ein Symptom von Anorexie sein.
4. Gene spielen bei der Entstehung von Essstörungen keine Rolle.
5. Essstörungen haben mit die höchste Sterblichkeitsrate aller psychischen Störungen.
6. Männer können eine Anorexie, aber keine Bulimie entwickeln.
7. Wenn Menschen sich frühzeitig Hilfe für eine Essstörung suchen, werden sie schneller wieder gesund.
8. Menschen mit Bulimie können leicht untergewichtig, normalgewichtig oder übergewichtig sein.
9. Die meisten Menschen haben schon einmal Essattacken gehabt.
10. Um an Bulimie zu leiden, muss man sich übergeben.
11. Häufig haben Menschen mit Essstörungen auch noch eine andere psychische Störung, wie z.B. Depressionen.
12. Menschen mit Essstörungen können mit ihrem Verhalten jederzeit aufhören wenn sie wollen.
13. Nur jugendliche Mädchen und junge Frauen sind von Essstörungen betroffen.
14. Nicht jeder der an einer Essstörung erkrankt, braucht Hilfe.
15. Essstörungen werden einzig durch ein in der westlichen Kultur anerkanntes Schlankheitsideal verursacht.
16. Ein rascher Gewichtsverlust oder starkes Untergewicht kann Auswirkungen auf die Denkfähigkeit haben.
17. Menschen mit Essstörungen könnten es schwierig finden, Familie oder Freunde um Hilfe zu bitten.
18. Menschen, die eine Essstörung hatten, werden sich immer Gedanken über ihr Gewicht machen, auch nach einer vollständigen Genesung.
